# A Mobile App to Enhance Awareness of Vaccination in Adults With Psoriasis and Atopic Dermatitis: Development and Preliminary Evaluation Study

**DOI:** 10.2196/80113

**Published:** 2025-12-09

**Authors:** Giulia Gasparini, Donatella Panatto, Irene De Kermarek, Lucia Valchi, Elvira Massaro, Piero Luigi Lai, Martina Burlando, Giancarlo Icardi, Emanuele Cozzani

**Affiliations:** 1 Department of Health Sciences University of Genoa Genoa Italy; 2 Dermatology Unit Ospedale Policlinico San Martino Genoa Italy

**Keywords:** vaccination, awareness, psoriasis, atopic dermatitis, mobile app, Mobile App Rating Scale

## Abstract

**Background:**

Patients with psoriasis and atopic dermatitis are at increased risk of several vaccine-preventable diseases. Despite this increased susceptibility to infections, vaccination uptake in adults with psoriasis and atopic dermatitis, especially if treated with biologics and other systemic immunomodulators, is insufficient. As mobile health technologies may support behavior change, a mobile app called DermatoVax was developed to raise awareness of immunization in adult patients with psoriasis and atopic dermatitis.

**Objective:**

This paper aims to describe the processes of development of the DermatoVax app and its initial evaluation in terms of technical verification and physicians’ quality rating.

**Methods:**

The app was conceived in a user-centered fashion. Its core component was the vaccine checker, which allows the app to produce a sharable list of recommended vaccines, immunization timings, and eventual precautions from a short set of input data. App prototypes were extensively piloted, and feedback from potential end users was obtained to refine the app content. The readability of the textual narratives was measured using the Italian-specific Gulpease index, which ranges from 0 to 100, where 100 indicates the best readability. The quality of the final version was evaluated by 46 medical doctors (n=29, 63% dermatologists and n=17, 37% public health physicians) using a validated Italian user version of the Mobile App Rating Scale (uMARS).

**Results:**

Iterative steps during the app development process allowed us to increase its user-friendliness and comprehensibility. Proper functioning of the checker was confirmed through the correct and complete generation of recommended vaccine lists for 50 mock patients with psoriasis and atopic dermatitis. An overall Gulpease index of 41.0 was observed for the final textual narratives, suggesting acceptable readability properties for patients with a high school diploma. Of a maximum of 5 points, the average uMARS score was 4.22 (SD 0.49). Ratings provided by dermatologists (mean 4.28, SD 0.48) were similar (*P*=.33) to those provided by public health physicians (mean 4.12, SD 0.51). However, the mean uMARS scores for the quality dimensions of aesthetics (3.88, SD 0.78) and engagement (3.89, SD 0.68) were lower than those for information (4.64, SD 0.42) and functionality (4.47, SD 0.46), suggesting margins for improvement. The app’s perceived impact was notably high, with over 80% of physicians agreeing that its use would significantly improve patient awareness (39/46, 85%) and knowledge (41/46, 89%) of vaccination, leading to increased vaccination uptake (37/46, 80%).

**Conclusions:**

DermatoVax is a promising tool to raise awareness of immunization in adult patients with psoriasis and atopic dermatitis. Further assessment of the app, such as its effectiveness in increasing vaccination uptake, is warranted.

## Introduction

Psoriasis and atopic dermatitis (AD) are 2 common chronic skin diseases that affect approximately 2% to 4% of the adult population worldwide [1,2]. Prevalence of adult AD in the overall population is generally lower among men; it decreases with age and shows some important geographic variation, ranging from 2.1% in Japan to 4.9% in the United States [2]. Similarly, the incidence of psoriasis varies from 78.9 per 100,000 person-years in the United States to 230 per 100,000 person-years in Italy [1]. Apart from a significant impact on physical, mental, and social well-being [3], patients with psoriasis and AD are also at increased risk of infections, including several vaccine-preventable diseases (VPDs). For instance, a large US cohort [4] showed that the incidence of herpes zoster (HZ) in adults with psoriasis (11.35 per 1000 person-years) was significantly higher than in adults without psoriasis (7.67 per 1000 person-years), with an adjusted incidence rate ratio of 1.21. Dermatological patients are also at an increased risk of postherpetic neuralgia even if the underlying disease is in remission [5]. Analogously, another US study reported [6] that patients with AD were much more likely (adjusted odds ratio 1.73, 95% CI 1.54-1.95) to develop pneumonia or contract influenza than patients without AD. The increased susceptibility to infections is driven by a complex interplay among disruption of the skin barrier; immune dysregulation; and environmental factors, including the use of immunosuppressants [7]. Indeed, both psoriasis and AD are T cell–mediated inflammatory diseases that can be treated with specific cytokine antagonists or more broad immunosuppressive drugs [8].

Despite the increased risk of VPDs, the available data suggest an insufficient vaccination uptake in adult patients with psoriasis and AD, especially in those of younger age. In a recent US survey of adult patients with AD and psoriasis or psoriatic arthritis treated with biologics and Janus kinase inhibitors [9], coverage rates for the seasonal influenza, HZ, pneumococcal, and COVID-19 vaccines were only 9.5%, 6.8%, 16.7%, and 63.9%, respectively. Analogously low coverage rates for the principal routine vaccines were noted among French patients with psoriasis [10]. While recognizing the individual and societal benefits of vaccines, patients with psoriasis and eczema believe that several barriers (such as lack of access to information about vaccines; lack of time for vaccine appointments; concerns about side effects, including disease flaring; and misinformation) contribute to low vaccination uptake among them [11]. Notably, according to Noe et al [12], influenza vaccine uptake in patients with psoriasis is associated with age, female gender, and some comorbidities but not with systemic treatment, including biologics. Another multinational study [13] showed that, compared with other treatment regimens, patients with psoriasis treated with biologics were much less likely to receive at least one COVID-19 vaccine dose. The latter findings may imply that a substantial proportion of patients with psoriasis and AD on systemic therapy with biologics or immunomodulators and with no other comorbidities are unaware of vaccines that they are recommended to receive or are hesitant to be immunized.

With the potential to reach large numbers of users, mobile health (mHealth) apps offer unique opportunities to deliver self-care interventions and support behavior change in a cost-effective way [14] and are increasingly common in all branches of medicine, including dermatology [15]. Indeed, hundreds of dermatology-specific apps focusing on disease guides, self-surveillance, and self-diagnosis or providing general dermatology reference and educational aid are available at app stores [15]. With regard to vaccines, mobile apps have been shown to raise disease-specific awareness on VPDs [16,17]; improve health care providers communication about vaccination [18]; and, to a lesser extent, increase vaccination uptake [19].

The insufficient vaccination coverage in patients with psoriasis and AD despite their increased risk of VPDs is a clear unmet need in this large population group. Low awareness is likely a major driver of the low vaccination adherence. In proof, three-fourths of German patients with psoriasis and AD reported that they had not been vaccinated against pneumococcal disease because of a lack of recommendation by a physician [20]. Furthermore, to the best of our knowledge, there are no unified Italian recommendations for comprehensive immunization schedules for adult patients with psoriasis and AD. Current guidance is fragmented, exemplified by the limited scope of disease-specific recommendations, such as those for the HZ vaccine [5]. This lack of a centralized resource complicates clinical decision-making for both patients and health care providers. Against this background, we recently developed and launched the DermatoVax mobile app and a companion website that specifically target Italian adults living with psoriasis and AD. The overarching aim of this project was to raise awareness of immunization and, hopefully, increase vaccination uptake among these patients. In this paper, we report the process of development, initial validation, and evaluation of the DermatoVax app.

## Methods

### Development of the DermatoVax App

The DermatoVax project was conceived as a way to increase knowledge and promote awareness of the importance of vaccination among adult (aged ≥18 years) patients with psoriasis and AD, with the ultimate goal of improving vaccination uptake in this target population group. The process of app development involved several iterative phases and considered our previous experience with the Pneumo Rischio app launched to increase awareness of invasive pneumococcal disease in the general population [16]. Indeed, both projects share several common aspects in their goals and features and were developed in a user-centered fashion.

To decide on the app content and features, a steering committee was first organized; it was composed of 3 dermatologists, 2 public health physicians, 1 specialist in communication, and 2 patient representatives. Following agreement on the scope, content, and features of the future app, an initial on-paper prototype was conceived and reviewed by the committee members. It was unanimously felt that the app should be maximally functional, highly intuitive, and user-friendly.

All textual narratives used on the app were assessed for their readability using the Gulpease index [21], which is specific to the Italian language. It considers the average number of words per sentence and the average number of letters per word. The index ranges from 0 to 100, where 100 indicates the best readability. We chose the target index value of ≥40 as texts with a Gulpease index of <40 are expected to be difficult to read for individuals with a high school diploma. Patient representatives reviewed the app content for comprehensibility, and based on their feedback, some small changes were made. A reconciled paper-based version was shared with a contracted IT company to create an iterative series of wireframes, which were used to test and optimize the overall design, schematic, pathways, and interactions.

An initial app prototype was then developed and piloted in several modalities. First, potential end users (medical residents, nurses, and administrative staff), who were all outside the research team (n=6; 3/6, 50% male and 3/6, 50% female; aged 26-66 years), were asked to navigate the app and perform 5 different technical tasks (to locate the privacy policy, perform a mock session using the vaccine checker, locate information on psoriasis, locate information on influenza vaccination, and identify the nearest vaccination center). Technical effectiveness was measured by means of the task completion rate, which is the proportion of successful attempts to the total number of attempts [22].

**Figure 1:**
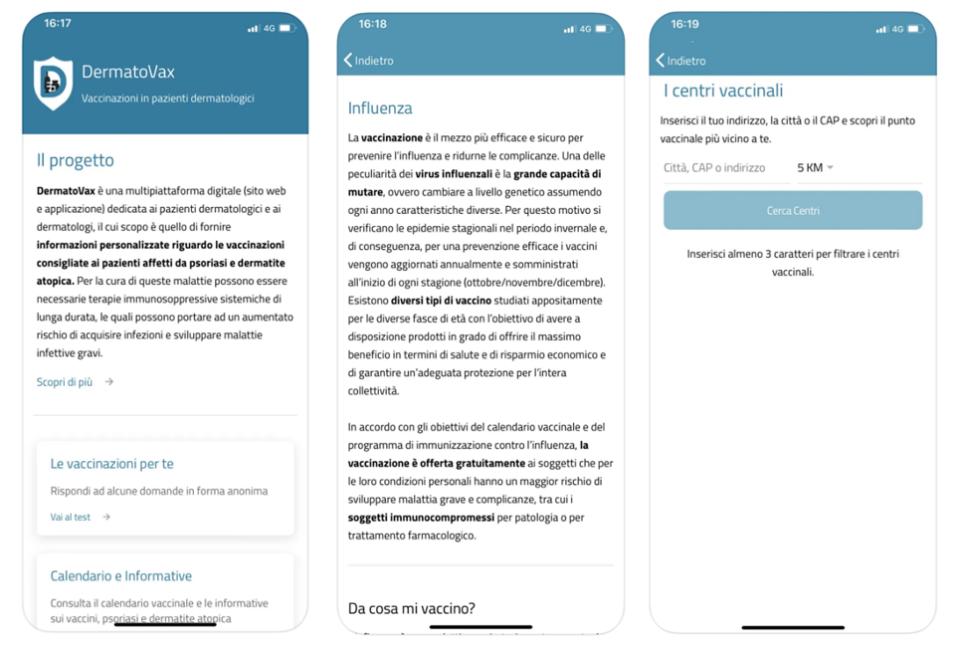
Representative screenshots of the DermatoVax app: (A) screenshot of the home page, with project explanation and links to the various parts of the app; (B) screenshot that shows an example of information on the influenza vaccine; and (C) screenshot of the vaccine center finder, where patients can find the closest vaccination center in a 5- or 10-km radius by typing their address.

Second, the computational algorithm was tested for eventual bugs by performing mock sessions, in which 50 hypothetical patients with psoriasis (n=25, 50%) and AD (n=25, 50%) of different ages, with different comorbidities, and on various treatment regimens completed the checker, which is the core app component (see the Content and Features of the DermatoVax App section), and the output produced was verified for correctness and completeness. Finally, individual usability sessions with 3 patients with psoriasis and 2 patients with AD were conducted. These patients were instructed to navigate through all app components, fill in the checker, download the resulting output report, and assess its clarity. The customer feedback was gathered, and eventual amendments were made. The final version of the app was released on the Apple App Store in May 2023 (Figure 1). A representative set of screenshots of the DermatoVax app is also provided in Multimedia Appendix 1.

### Content and Features of the DermatoVax App

The DermatoVax app consists of 4 main sections. The first section provides a brief description of the project and a periodically updated news feed. The app clearly identifies its owner and developer and the project funder and provides contact details, bibliographic references, terms and conditions, a privacy policy, and links to principal institutional websites.

The second section is purely informative and provides essential information on psoriasis, AD, recommended vaccines, and the associated VPDs. The Italian National Immunization Plan [23] was the primary normative document used to identify the recommended vaccines according to the age group and risk factors. As no specific Italian guidelines for vaccination of patients with psoriasis and AD are available, and given the fact that there is a large overlap between immunosuppressive and immunomodulating drugs for the treatment of psoriasis or AD and rheumatic diseases, we then consulted the recommendations for vaccination of patients with rheumatic diseases issued by the Italian Society of Rheumatology [24]. Indeed, while the Italian National Immunization Plan [23] is intended as a general reference document, the Italian Society of Rheumatology recommendations [24] provide a comprehensive assessment (including grading of the quality of evidence and strength of recommendations) of each inactivated or live attenuated vaccine also in relation to the timing of vaccine administration during immunosuppressive and immunomodulating therapy with the most common drugs. In patients with psoriasis and AD, vaccines may be indicated for 3 main reasons (due to age; the presence of comorbidities such as chronic cardiovascular, respiratory, and other conditions; or the immunosuppressive treatment regimen).

The core part of the app is a vaccine checker, which allows for the creation of a list of recommended vaccines from a short set of input questions. As shown in Table 1, the checker considers patients’ nosological entity, eventual hand involvement, age, current or upcoming chronic treatment with 1 of 25 common drugs and their dosing, and presence of comorbidities and other risk factors. Upon informed consent, the checker is filled in, and an output report is produced. The latter summarizes patients’ inputs and provides a detailed list of the recommended vaccines. In particular, the following vaccines were considered: diphtheria, tetanus, and acellular pertussis; *Hemophilus influenzae* type B; hepatitis B; hepatitis A; measles, mumps, and rubella; varicella; HZ; quadrivalent meningococcal vaccine; meningococcal B vaccine; conjugated and 23-valent polysaccharide pneumococcal vaccines; seasonal influenza; COVID-19; human papillomavirus; and mpox. Each vaccine-specific recommendation is accompanied by a brief description of vaccination timing in relation to the current or planned treatment (eg, according to the medicine used, live attenuated vaccines should not be administered for a certain period, whereas inactivated vaccines may be generally administered during the immunosuppressive therapy, although their administration is preferable at least 2 weeks before treatment initiation), boosting (eg, a diphtheria, tetanus, and acellular pertussis vaccine booster dose every 10 years), and seasonality (eg, administration of influenza and COVID-19 inactivated vaccines). The list of recommended vaccines may be saved and shared with the patients’ dermatologists, general practitioners (GPs), or other health care providers. Patients are warned that the report has an informative character only and are invited to refer to their immunization records and discuss vaccination strategies with their dermatologists or vaccinating physicians. As the algorithm does not consider eventual risks of professional exposure or travel intentions, patients are informed that other vaccines may also be recommended, information on which may be obtained from health care providers.

Finally, the app allows users to identify the nearest vaccination centers and offers links to institutional websites where users can find further relevant information on vaccines, including possible adverse events and their reporting procedures.

**Table 1:** Items and response options of the vaccine checker used in the DermatoVax mobile app to produce a list of recommended vaccines.

Item	Response options
Disease	“Psoriasis” and “atopic dermatitis”
Hand involvement	“Yes” and “no”
Age	“18-59 years,” “60-64 years,” and “65 years or more”
Current or upcoming chronic (>2 weeks) therapy	“Yes” and “no”
Medication prescribed (if the previous item was answered with “yes”)	“Abrocitinib,” “acitretin,” “adalimumab,” “apremilast,” “baricitinib,” “bimekizumab,” “brodalumab,” “certolizumab pegol,” “ciclosporin,” “dimethyl fumarate,” “dupilumab,” “etanercept,” “golimumab,” “guselkumab,” “infliximab,” “ixekizumab,” “methotrexate,” “prednisone,” “risankizumab,” “secukinumab,” “tildrakizumab,” “topical medications (cremes, ointments, etc.),” “tralokinumab,” “upadacitinib,” and “ustekinumab”
Ciclosporin daily dose (if the previous item was answered with “ciclosporin”)	Dose in milligrams
Methotrexate weekly dose (if the previous item was answered with “methotrexate”)	Dose in milligrams
Prednisone daily dose (if the previous item was answered with “prednisone”)	Dose in milligrams
Weight (if the previous item was answered with “ciclosporin” or “methotrexate”)	Weight in kilograms
Health conditions or other lifestyle characteristics	“Type 1 diabetes,” “type 2 diabetes,” “respiratory diseases,” “cardiovascular diseases,” “congenital or acquired immunodeficiency,” “HIV infection,” “chronic kidney failure/renal disease,” “asplenia,” “chronic hepatic disease or alcoholism,” “man who has sex with men,” and “pregnancy”

### External Evaluation of App Quality

To evaluate the quality of the DermatoVax app, a convenience sample of board-certified dermatologists and public health physicians were enrolled during 3 local conferences where the project was presented. All physicians were outside the research team and had been unaware of the project. In particular, following a presentation of the app, the conference audience (approximately 80-120 attendees per conference) was asked to download the app (<2 minutes), navigate all its components and functions for at least 10 minutes, and fill in the previously validated Italian user version of the Mobile App Rating Scale (uMARS) [25]. Contrary to the professional version of the Mobile App Rating Scale (MARS) [26,27], the uMARS is simpler and does not require preliminary training [28]. The tool consists of 4 objective quality subscales, namely, engagement, functionality, aesthetics, and information, which comprise a total of 16 items. There are also 2 additional subscales that measure subjective quality (4 items) and perceived impact (6 items) of the evaluated app. Each of the 26 items is rated on a 5-point Likert scale, where a greater score corresponds to a better quality. Scores of individual items are averaged to obtain a mean quality score for each objective subscale, whereas the resulting 4 mean subscale scores are averaged to obtain a uMARS total score. The Italian version of the uMARS has an excellent internal consistency (Cronbach α=0.94) [25]. The questionnaire was administered anonymously using a pencil-and-paper format.

With an assumed SD of the total uMARS score of 0.45 [29], precision of 0.15, and confidence level of 95%, at least 35 physicians were needed to estimate the population mean.

### Data Analysis

Total, subscale, and item-specific uMARS scores were summarized as means with SDs and medians with IQRs. Two-tailed independent *t* tests were used to compare uMARS scores between dermatologists and public health physicians. Data analysis was conducted in the R *stats* packages (version 4.1.0; R Foundation for Statistical Computing) and Microsoft Excel.

### Ethical Considerations

This study was conducted in accordance with the Declaration of Helsinki of 1964 and its later amendments. The study protocol was approved by the Ethics Committee of Liguria Region, Italy (583/2021; ID 11870). Informed consent was obtained from all participants. All uMARS questionnaires collected were anonymous. No compensation was provided to either patients or health care professionals.

## Results

### Pilot-Testing of the DermatoVax App

During the piloting of the app prototype, no major changes were deemed necessary. In particular, all 6 testers succeeded in performing the 5 tasks with a task completion rate of 100% (30/30 tasks), and the task completion time was deemed acceptable (range 1-4 min). However, some minor changes regarding navigational components and shortcuts were suggested. The output files with a list of recommended vaccines generated from the input data of 50 mock patients were deemed correct and complete, suggesting a proper functioning of the algorithm.

During the individual usability sessions with 5 real patients, the checker was completed within 6 minutes (range 2-6 min). The feedback obtained from the patients was positive, and no changes were deemed necessary to any app component. In the final app version, the average readability index of the textual narratives was 41.0.

### Quality Scores and Perceived Impact of the DermatoVax App

A total of 46 physicians (n=29, 63% dermatologists and n=17, 37% public health physicians) filled in the uMARS tool (Table 2). Their median age was 47 (IQR 38-59) years, and approximately two-thirds (29/46, 63%) were female. The average uMARS score was 4.22/5 (SD 0.49), with a range of 2.91 to 5.00. The mean scores were similar (*P*=.33) between dermatologists (mean 4.28, SD 0.48) and public health physicians (mean 4.12, SD 0.51). An analysis by single objective subscale revealed that the average scores for the information (4.64/5, SD 0.42) and functionality (4.47/5, SD 0.46) dimensions were comparatively high, whereas those for the dimensions of aesthetics (3.88/5, SD 0.78) and engagement (3.89/5, SD 0.68) were substantially lower.

Regarding the subjective quality dimension, the average score of 4 items was 3.59 (SD 0.51). The score was mainly determined by the “Would you pay for this app?” item: only 39% (18/46) of physicians replied affirmatively (“yes” or “definitely yes”), and the average score was only 2.74 (SD 1.44). Conversely, most responders would recommend the DermatoVax app (36/46, 78%) and use it at least 10 times in the following 12 months (31/46, 67%). The average star rating (out of 5 stars) was 3.87 (SD 0.65).

**Figure 2:**
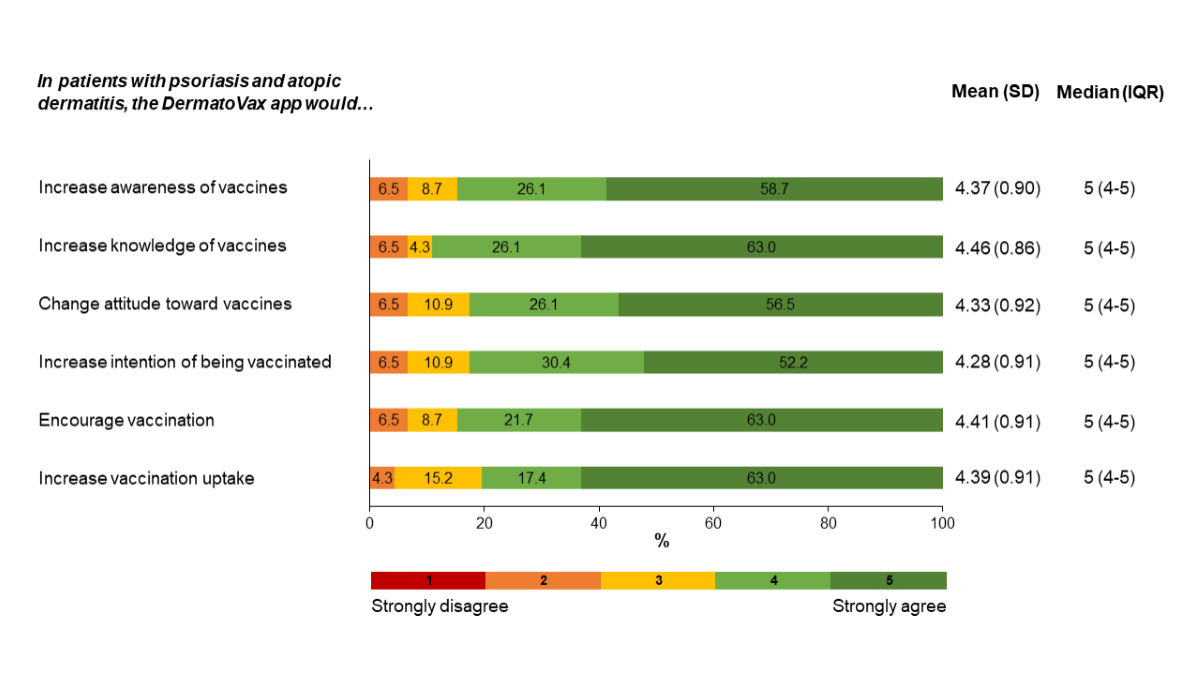
Physicians’ perceived impact of the DermatoVax app on vaccination-related knowledge, attitudes, and practices (N=46).

Finally, we investigated physicians’ perceived impact of the DermatoVax app on vaccination-related knowledge, attitudes, and practices among patients with psoriasis and AD. As shown in Figure 2, the perceived impact was high: at least four-fifths of physicians agreed or strongly agreed that use of the app would increase patient awareness (39/46, 85%) and knowledge (41/46, 89%) of vaccines and vaccination uptake (37/46, 80%).

**Table 2:** User version of the Mobile App Rating Scale (uMARS) scores from physicians who evaluated the DermatoVax mobile app by objective subscale and item (N=46).

			Score, mean (SD)		Score, median (IQR)
**Engagement subscale**					
	Entertainment	3.83 (0.77)		4 (3-4)	
	Interest	4.33 (0.67)		4 (4-5)	
	Customization	3.50 (1.15)		4 (3-4)	
	Interactivity	3.52 (0.98)		4 (3-4)	
	Target group	4.26 (0.74)		4 (4-5)	
	Total	3.89 (0.68)		4.00 (3.40-4.20)	
**Functionality subscale**					
	Performance	4.33 (0.67)		4 (4-5)	
	Ease of use	4.67 (0.47)		5 (4-5)	
	Navigation	4.46 (0.66)		5 (4-5)	
	Gestural design	4.41 (0.62)		4 (4-5)	
	Total	4.47 (0.46)		4.50 (4.25-4.75)	
**Aesthetics subscale**					
	Layout	4.09 (0.81)		4 (4-5)	
	Graphics	3.78 (0.92)		4 (3-4)	
	Visual appeal	3.78 (0.76)		4 (3-4)	
	Total	3.88 (0.78)		4.00 (3.33-4.33)	
**Information subscale**					
	Quality of information	4.74 (0.49)		5 (5-5)	
	Quantity of information	4.52 (0.62)		5 (4-5)	
	Visual information	4.54 (0.55)		5 (4-5)	
	Credibility of source	4.80 (0.55)		5 (5-5)	
	Total	4.64 (0.42)		4.75 (4.38-5.00)	
uMARS total score			4.22 (0.49)		4.29 (3.98-4.49)

## Discussion

### Principal Findings

In this paper, we described an initial evaluation of DermatoVax, the first mobile app to increase awareness of vaccines and VPDs that was specifically developed for patients with immune-mediated inflammatory skin diseases. The advent of novel immunomodulatory and biological treatments has revolutionized the treatment of psoriasis and AD. At the same time, these new-generation therapies require particular considerations regarding infections and immunization [30]. Indeed, the recommended vaccination schedule for patients with psoriasis and AD is rather complex and nuanced and depends on age, presence of comorbidities, treatment regimen, and other factors [24,30,31]. This complexity may be burdensome for both patients and health care workers and can lead to reduced vaccination adherence [32]. We believe that mHealth projects such as DermatoVax may empower patients and dermatologists to navigate the complex vaccination landscape. Indeed, most participating dermatologists and public health physicians believed that the DermatoVax app can raise awareness of vaccines (39/46, 85%) and increase vaccination uptake (37/46, 80%) in patients with psoriasis and AD.

The initial validation and individual usability sessions showed that the DermatoVax mobile app was valued by potential end users, who judged it as intuitive, user-friendly, and potentially helpful. Contrary to traditional immunization schedules, which often have numerous and complex footnotes [33], personalized reports on recommended vaccines generated by the app were deemed easily interpretable. Chen et al [34] suggest that human factors can be implemented to improve the usability of adult immunization schedules; the application of some design principles, such as consistency, simplicity, and clarity, enhances schedule efficiency while maintaining a form that fits better with user expectations of the schedule.

The quality of mHealth apps is a multidimensional concept that defines user experience [26]. The MARS [26] and uMARS [28] tools are currently the most widely used scales for evaluating content and the overall and domain-specific quality of mHealth apps. Indeed, the content of a health-related app may not accurately reflect its description and features [26]. An Italian study [35] identified 121 vaccine-related apps, only 32% of which provided some information on vaccines or VPDs. In our study, the mean uMARS scores given by physicians (4.22, SD 0.49) were relatively high. In comparison, the average MARS scores of 21 immunization-related apps available in Chinese app stores is 3.33 (SD 1.07) [36]. A German evaluation of 8 apps focused on psoriasis yielded MARS scores ranging from 3.00 to 4.18 [37]. However, it should be noted that MARS and uMARS scores may be not fully comparable; some studies [37,38] have suggested that mean uMARS scores given by patients may be slightly (0.3 points) lower than average MARS scores given by physicians. This fact underlines the importance of patient involvement in all steps of the app development and validation process.

In evaluating single dimensions of app quality, we found that the mean information (4.64, SD 0.42) and functionality (4.47, SD 0.46) uMARS subscales received the highest scores, whereas the engagement (mean 3.89, SD 0.68) and aesthetics (mean 3.88, SD 0.78) subscales were associated with lower scores. This differential scoring pattern clearly highlights the distinction between the app’s utility and its appeal. A similar pattern has been reported in previous studies on the quality of vaccine- [36] and psoriasis-related [37] apps. This finding was expected because the DermatoVax app is intrinsically bound to be scientifically sound and computationally simple and, therefore, highly informative and functional. On the other hand, an improved functionality and minimalist design may have compromised some engagement attributes and the overall aesthetic perception of the app. In other words, the focus on streamlined utility can clash with consumer expectations for visually rich and interactive apps. This, in turn, could eventually lead to low retention rates (ie, when users delete the app after a few sessions). Future developments of the DermatoVax app will prioritize enhanced engagement and refined aesthetics. This could be achieved through a comprehensive user interface and user experience redesign, the integration of additional gamification elements, and advanced personalization features and feedback elements. In particular, the user interface refinement would ensure a visually polished, modern, and intuitive appearance, whereas the user experience redesign would further streamline the navigation flow, making the latter even less effortful. Implementation of more advanced features that tailor content delivery based on the patient’s specific clinical profile (eg, previous vaccines) will further enhance app personalization. Finally, the introduction of dynamic feedback loops would improve long-term user retention.

We then found that only one-third of physicians (18/46, 39%) would purchase the app, which suggests that the willingness to pay for apps such as DermatoVax is likely low. We found a similar proportion when evaluating the Pneumo Rischio mobile app [29], which has several similarities to the DermatoVax app. This finding indicates that, to reach a larger number of end users, such “niche” and prevention-oriented apps should be accessible to everyone at no cost.

To our knowledge, there are no data on vaccination uptake (of any vaccine) among Italian patients with immune-mediated inflammatory skin diseases. Considering the available data from international surveys [9,10], we believe that immunization rates among Italian patients are similarly low. In their pilot work, Feldman et al [39] highlighted numerous unmet preventive health care needs, including vaccination, among patients attending a dermatology clinic and discussed ways of improvement. In particular, one approach could consist of more comprehensive general preventive care provided by dermatologists. However, as noted by the authors, even if dermatologists were specifically trained, this possibility may compromise the time efficiency of dermatological services. Another approach, which seems more promising, consists of a cooperation between dermatologists and primary care physicians: the former may actively identify patients with unmet preventive care needs and refer those patients directly to GPs or other preventive medicine specialists [39]. We believe that mHealth tools such as DermatoVax may facilitate continuity of care, enabling interdisciplinary teamwork that puts patients’ need first. Indeed, some research suggests [20] that dermatologists are rarely involved in vaccination counseling. One recent study [40] found that 44% of patients with psoriasis searched the web for additional information as the information provided by their physicians was insufficient. At the same time, 45% wanted their physicians to recommend health-related websites to them. In this regard, dermatologists may recommend their patients free and user-friendly apps to check their vaccination status and eventually receive missing recommended vaccines from their GPs or public health physicians.

This study represents a preliminary evaluation and, as such, is subject to several important limitations. First, external evaluation of the DermatoVax app and its quality was conducted by dermatologists and public health physicians only. Therefore, the most important shortcoming of this study is the lack of formal quality evaluation of the app by patients. Analogously, data on actual app use, and especially its ability to change vaccination status, are yet to be collected and assessed. The app ratings obtained from health care professionals may be systematically different from those provided by patients, who are supposed to be the main app audience. For instance, in evaluating an app, patients could prioritize features such as usability, empowerment, and self-sufficiency, whereas physicians could prioritize clinical aspects such as the use of evidence-based practices and guidelines. More importantly, health-related apps should ideally be trialed for their efficacy or effectiveness. However, only a few mobile apps have undergone formal evaluation of their effectiveness [26]. As underlined by the available systematic reviews [41,42], data on the efficacy and effectiveness of mobile apps and other digital technologies on actual vaccine receipt are limited, heterogeneous, and inconclusive, with some studies showing significant benefits and other trials reporting no effect. To address this limitation, we aim to conduct a larger and more comprehensive evaluation of the DermatoVax app to determine both its quality from the end user perspective and its capacity to modify the immunization knowledge, attitudes, and practices of patients with psoriasis and AD. Second, individual usability sessions were conducted with 5 patients with psoriasis and AD as, according to the “5-user rule,” only 5 participants are needed to discover 80% of usability problems [43]. However, we recognize that this number may be small for a comprehensive understanding across diverse patient profiles or for uncovering less frequent but critical issues. Third, owing to the cross-sectional nature of this study without longitudinal follow-up, our findings reflect only a single snapshot in time. Finally, as the app was developed and evaluated solely in Italian, its current utility is restricted to Italian speakers, raising questions about its generalizability across diverse cultural settings and non–Italian-speaking populations such as immigrants.

Importantly, immunization strategies and recommendations, available vaccines, and biological therapies are constantly evolving. This dynamic landscape underscores the critical importance of keeping the app content current. For instance, respiratory syncytial virus vaccines for adults have recently become available [44]. Although these vaccines are not yet part of the Italian vaccination calendar and are not reimbursed, some Italian scientific societies have advocated for the inclusion of respiratory syncytial virus vaccines in the national vaccination schedule [44]. Hence, our commitment to app maintenance involves periodic updates to consistently include these novel advancements.

### Conclusions

In conclusion, this preliminary assessment showed that the DermatoVax mobile app is a promising tool to raise awareness of immunization and VPDs in adult patients with psoriasis and AD, whose uptake of the recommended vaccines is insufficient. Positive feedback of end users and good quality scores of the app from health care providers merit further evaluation, in particular of whether app use may effectively change vaccination-related behaviors. Furthermore, continuously evolving adult immunization schedules [33] and clinical management protocols for immune-mediated inflammatory skin diseases [45] impose the necessity of keeping the app content constantly up-to-date.

## Appendix

Multimedia Appendix 1 Representative set of screenshots of the DermatoVax app developed to raise awareness of immunization in adult patients with psoriasis and atopic dermatitis.

## Abbreviations

AD: atopic dermatitis
GP: general practitioner
HZ: herpes zoster
MARS: Mobile App Rating Scale
mHealth: mobile health
uMARS: user version of the Mobile App Rating Scale
VPD: vaccine-preventable disease

## Data Availability

The datasets generated or analyzed during this study are available from the corresponding author on reasonable request.
