# Supplementary figures and images for: A Mobile App to Enhance Awareness of Vaccination in Adults With Psoriasis and Atopic Dermatitis: Development and Preliminary Evaluation Study
Source: JMIR Form Res. 2025 Dec 9;9:e80113. doi: 10.2196/80113 (PMC12728400; doi:10.2196/80113)

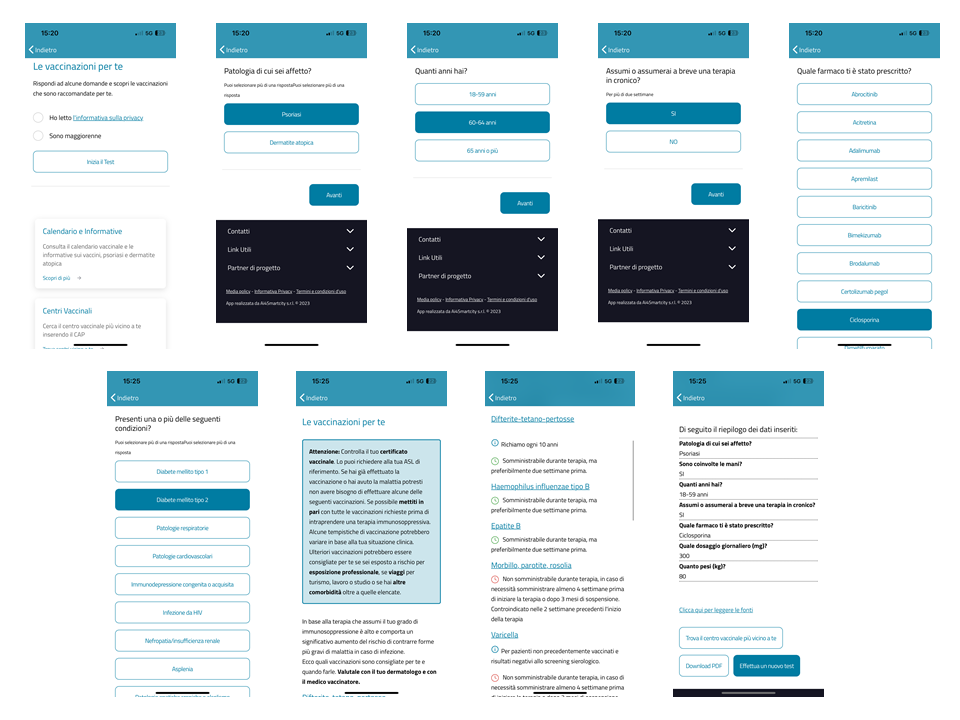

Supplement: Multimedia Appendix 1 [file formative_v9i1e80113_app1.png]
